# Influence of genetic background and dietary oleic acid on gut microbiota composition in Duroc and Iberian pigs

**DOI:** 10.1371/journal.pone.0251804

**Published:** 2021-05-20

**Authors:** Adrián López-García, Rita Benítez, Yolanda Núñez, Emilio Gómez-Izquierdo, Eduardo de Mercado, Juan M. García-Casco, Óscar González-Recio, Clemente López-Bote, Jordi Estellé, Cristina Óvilo

**Affiliations:** 1 Department of Animal Breeding, INIA, Madrid, Spain; 2 Pig Test Center ITACYL, Hontalbilla, Segovia, Spain; 3 Department of Animal Production, Veterinary Faculty, UCM, Madrid, Spain; 4 GABI, INRAE, AgroParisTech, Université Paris-Saclay, Jouy-en-Josas, France; University of Bologna, ITALY

## Abstract

**Background:**

Phenotypic variability for productive and meat quality traits has been largely studied in Iberian pigs, especially in genetic selection and nutritional experiments. Complex interactions among genetic background, diet composition and gut microbiota hinder the correct assessment of each factor’s contribution on phenotypes. In order to disentangle these interactions, we evaluated changes in gut microbiota composition comparing 48 Iberian and Duroc pigs fed diets with different energy source (standard diet with carbohydrates vs sunflower oil-enriched diet with high oleic acid content).

**Results:**

A higher richness was observed for Iberian pigs (p < 0.05) and compositional analysis was applied for beta-diversity, differential abundance and pairwise log-ratio analyses. We found significant differences in overall microbiota composition between breeds, and also between diets inside breeds, to a lesser extent. Differential abundance analysis revealed that Duroc animals have more proportion of Actinobacteria and *Prevotella*, while Iberian replace those microorganisms with other more variable taxa. According to dietary differences, high-oleic fed animals were richer in *Prevotella*. We also found microbial ratios capable of separating animals by breeds and diets, mostly related to Actinobacteria.

**Conclusion:**

This study reveals that both genetic background and diet composition might have a relevant impact in gut microbiota composition. The application of compositional data analysis has facilitated the identification of microorganisms and ratios as possibly related to metabolic changes due to genetic background and, to a lower extent, to dietary changes. This may lead to a relevant progress in the knowledge of interactions between pig genetics, environment and gut microbiota.

## Introduction

The contribution of Spanish pig production to worldwide pork market is meaningful. According to FAOSTAT, Spain produces almost 4% of worldwide swine meat, being the fourth producer, and the second one inside EU, with a 19% of communal production, after Germany. One of the most eye-catching contribution of Spanish pig production to international markets comes from Iberian pig, a local breed whose high-quality final products are much appreciated. In fact, Spain ranks as the third country in lard and ham exportations (data from 2018) [1], with a crucial contribution of dry-cured Iberian ham. The high value of this product is due to the unique organoleptic properties conferred by Iberian pig genetic, phenotypic and production particularities. This rustic and lowly-prolific local breed has been traditionally raised under a semi-extensive production system in the “Dehesa” ecosystems, taking advantage of the grass and acorn resources available [2]. The combination of their natural high tendency to fat accumulation and the acorn and grass feeding in the late fattening phase (“Montanera”), confers their final products a unique fatty acid (FA) profile composition and a higher intramuscular fat (IMF) content than commercial breeds.

The interest in this high-quality market has led to the search and legal regulation of strategies for the improvement of its efficiency and productivity. In this sense, one of the most common practices nowadays is crossbreeding with Duroc, resulting in a negative impact on final product composition and quality [3]. Crossbreeding consequences have been largely studied through different points of view—such as phenotypic traits, meat quality, genetic markers or gene expression [4–6]. As the high quality of Iberian products has become the flag for the Iberian pig market to enter international markets, producers keep an interest in improving their products’ organoleptic attributes through FA profile and IMF content optimization [7, 8], especially in the recent more intensive production systems. Several strategies such as genetic selection, handling optimization and diet design, are methods of modifying final product composition. Strategies such as dietary FA supplementation are common to enhance these organoleptic traits. Genetic, nutrition and nutrigenomic studies have shown the complexity of the molecular regulation of physiological processes associated to relevant phenotypic traits in Iberian pig genotypes subjected to different diets [4, 9–11].

Besides, with the advent of new mass-sequencing techniques, alternative ways have been opened for animal breeding and for in-depth studies of the molecular basis of phenotypic variability. Microbiota studies have hugely benefitted from these NGS techniques, as culture-free sequencing allows obtaining a lot more information from microbiome communities. Gut microbiota has become a key focus in animal breeding industry, as well as in human health, since gut microbiota and host are linked in a bidirectional way; first, diet composition and other environmental elements, as well as animal genetics, might modify microbial population structure [12]; second, microbiota has been reported to have an impact on animal physiology, on multiple animal productive traits and also on the final products’ quality and composition [13, 14]. This intricate correlation network makes gut microbiota composition a key factor for understanding the relationships between genotype, phenotype and environment. As a consequence, it might result obvious that different genetic backgrounds can promote different microbiota population structures as it has been proven in other studies [15]. For this reason, the two main breeds employed in Iberian pig production (Iberian and Duroc) might have differences in gut microbiota composition possibly affecting relevant phenotypic traits. Also, dietary modification or supplementation strategies such as FA addition might have an impact on gut microbiota composition, potentially contributing to differences in animal traits, with special relevance on final product composition and quality. Understanding all these relationships and their implications is crucial to comprehend their global effect on animal phenotype.

In this study we compared the gut microbiota composition in pigs from Iberian and Duroc breeds, fed two different isocaloric and isoproteic diets with different energy sources (oleic acid vs carbohydrates), in order to dissect the diet and genetic background effects on the gut microbiota composition, as well as to deepen in the metabolic differences between breeds and diet groups, potentially mediated by gut microbiota.

## Methods

### Experimental design and sampling

The experiment was carried out at the experimental facilities of the Instituto Tecnológico Agrario de Castilla y Leon (ITACYL) Pig Test Center (Hontalbilla, Segovia, Spain). A total of forty-eight castrated male purebred pigs, 19 Duroc (DU) and 29 Iberian (Torbiscal strain) [16] (IB), were raised in the same commercial farm (IBERPEX S.L., Guareña, Badajoz, Spain), weaned at 28 days old and transferred to the experimental facility one month after weaning. The animals were kept under identical management conditions, housed in batches of 4 pigs/pen (1 m^2^ pig^-1^), with a concrete floor and straw bedding. Temperature was controlled at a mean of 23.8 °C throughout the experiment. At 70 days old (±2 days) and with a mean body weight of 19.7 ± 3.6 kg, both breeds were distributed in two different experimental groups with a factorial 2x2 design, and fed a control commercial diet with carbohydrates as energy source (C) (9 DU and 13 IB) or a sunflower oil-enriched diet with high oleic acid content (6%) (O) (10 DU and 16 IB) (Initial weights per group: 17.3 ± 2.1 kg BW in DU-C; 17.3 ± 3.5 kg BW in DU-O; 20.5 ± 2.8 kg BW in IB-C; 22.0 ± 3.5 kg BW in IB-O, these differences not being statistically significant). Both diets were formulated according to FEDNA nutritional guidelines (2006) using the Brill Formulation software (Brill Co., Georgia, USA) to be isocaloric and isoproteic (3.3 kcal digestible energy and 15.6% crude protein) (S1 File) and were provided *ad libitum*. Diets were provided for 47 days, until animals reached 50.4 ± 7.8 kg BW (Final weights per group: 53.2 ± 6.4 kg BW in DU-C; 49.0 ± 10.8 kg BW in DU-O; 48.7 ± 7.4 kg BW in IB-C; 51.3 ± 6.8 kg BW in IB-O, these differences not being statistically significant). Fresh water was provided *ad libitum*, with two drinking troughs available in each pen.

Animals were slaughtered in the same experimental facilities at the end of treatment, at 117 days old (±2 days), and individual stool samples were then collected from rectum and rapidly frozen on liquid N_2_ and preserved at -80°C until its use for microbiome analysis. All experiments were performed in accordance with the regulations of the Spanish Policy for Protection of Animals employed in Research and other scientific purposes RD53/2013, which meet the European Union Directive 2010/63/EU on the protection of animals used in experimentation. The project was approved on March 20, 2015, by the Comunidad de Madrid animal welfare and protection committee (reference number PROEX-007/15).

### DNA extraction and 16S sequencing

Microbiota DNA was extracted from the 48 stool samples using QIAamp PowerFecal^®^ kit (QIAGEN, Hilden, Germany) according to manufacturer’s standard protocol. Illumina MiSeq^®^ paired-end sequencing protocol (Illumina, San Diego, CA, USA) was performed by an external service (FISABIO bioinformatics, Valencia, Spain) targeting 16S rRNA gene V3-V4 amplicon. The employed primers were S-D-Bact-0341-b-S-17 and S-D-Bact-0785-a-A-21 [17], which produce amplicons of 464 bp. Raw microbial sequence data have been uploaded to the ENA repository and are available at https://www.ebi.ac.uk/ena/browser/view/PRJEB42303.

### Sequence processing pipeline

Raw reads were pre-processed using Prinseq-lite tool [18] and in-house software. After trimming low quality bases in each read, sequences shorter than 50 bp and with an average quality score below 30 on a window of 20 bases were discarded. After joining forward and reverse files, a total amount of 11,599,350 reads were processed using Quantitative Insights Into Microbial Ecology (QIIME) version 1.9.1 [19] into operational taxonomic units (OTUs), following the *de novo* OTU-picking approach, with a clustering threshold of 97% identity. Chimeras were filtered using USEARCH algorithm (v. 6.1) [20]. SILVA reference database (release 132) [21] was used for taxonomic assignment and chimera filtering. An OTU abundance threshold of 0.005% was established for final quality-filtering, as described in Bokulich *et al*. [22]. Processed OTU table was composed by 9,428,777 reads from 1,398 OTUs.

Finally, two additional processing steps were performed: a taxonomy filtering, excluding OTUs not assigned to Bacteria at kingdom level in the taxonomic classification, as used primers are designed for bacterial amplicons; and a sample pruning, removing those with sequencing depth lower than 30k total reads, which eliminated one sample for the IB-O group. The final OTU table was composed by 9,162,809 reads from 1,246 OTUs and 47 samples.

### Microbial community analysis

All analyses were performed in R, using the following packages: ALDEx2 [23], DESeq2 [24], easyCODA [25], limma [26], mixOmics [27], phyloseq [28], vegan [29] and zCompositions [30]. Microbiota composition was compared between breed-diet animal pens. Three different approaches were then followed to analyse microbiome composition:

#### Alpha-diversity

For this approach, OTU table was rarefied to minimum sample depth (30,136 reads). Four alpha-diversity measures were calculated: observed richness, Chao1 index, Shannon index and Inverse-Simpson index, using the function estimate_richness from phyloseq. ANOVA for each alpha-diversity measure was carried out following this model:

$$\alpha_{ijk}=\mu +b_{i}+d_{j}+{bd}_{ij}+e_{ijk}$$

Being *α* the alpha-diversity index for each sample, *b* the breed effect (with *i = 2* levels), *d* the diet effect (with *j = 2* levels) and *bd* the interaction of both effects.

#### Data transformation: Compositional data analysis

Most of the statistical analysis were made taking into account the compositional nature of microbiome data [31]. For the compositional approach our non-rarefied dataset was processed following a number of steps as defined by [32]: (1) Zero-counts imputing by Geometric Bayesian Multiplicative replacement; (2) Data closure computed to the total counts per sample; (3) Weighted centred log-ratio (CLR) transformation. This process moves the data to an additive scale, which makes the performance of multivariate hypothesis testing easier. The analysis was repeated glomming the dataset to OTU, genus, family and phylum levels. R packages zCompositions and easyCODA were used for this transformation.

#### Beta-diversity

Beta-diversity was computed using a compositional Principal Component Analysis (i.e., PCA after CLR transformation of the data). For a better visualization of the behaviour of both breed and diet effects, a mixed “breed-diet” factor was considered as PCA grouping variable. The distance matrix was built using Aitchison distance measure, which is the Euclidean distance after CLR transformation. Individual beta-diversities were calculated as the distance to centroid for groups from mixed “breed-diet” effect (using vegan::betadisper). Homogeneity of dispersions respect to the group centroids was measured through ANOVA-like permutation test (vegan::permutest) and post-hoc Tukey HSD test. For testing the significance of multivariate effects in our *a priori* groups, non-parametric permutational multivariate analysis of variance (PERMANOVA) [33, 34] was performed using adonis2 function from vegan package with this model:

$$D_{ij}=\mu +b_{i}+d_{j}+{bd}_{ij}+e_{ij}$$

The additive model applied was built sequentially adding breed (*b*_*i*_; *i = [1,2]*) and diet (*d*_*j*_; *j = [1,2]*) factors and their interactions (*bd*_*ij*_). The Aitchison distance matrix (*D*) was used as dependent variable.

### Differential abundance analysis

Differential abundance analysis was carried out using both DESeq2 and ALDEx2 R packages. Default DESeq2 normalization by estimation of size factors as the median ratio of counts [35] and negative binomial GLM fitting with Wald significance tests were performed. Differences between breeds and between diets were analysed in separate models correcting by the other factor. Interactions between factors were also evaluated with an additional model (Table 1). P-values were corrected for multiple testing through Benjamini-Hochberg method. Significantly differentially abundant (DA) OTUs were considered with a false discovery rate (FDR) cut-off of 0.05 and a fold-change (FC) higher than 1.5 or lower than -1.5 (i.e., |log2FC| > 0.59).

**Table 1:** DESeq2 differential abundance models for each contrast, with R notation.

Contrast	Model design
Breed contrast	*RA ratio* ~ *Diet* + *Breed*
Diet contrast	*RA ratio* ~ *Breed* + *Diet*
Interaction effects	*RA ratio* ~ *Breed* + *Diet* + *Breed*: *Diet*

Input for ALDEx2 analysis was prepared as the CLR-transformed posterior distribution of the data generated by 128 Monte Carlo samples from the Dirichlet distribution. CLR transformation was made through geometric mean of all features’ abundance. Mono-factorial breed and diet contrasts were performed, using Welch’s t-test. P-values were corrected through Benjamini-Hochberg method. OTUs were considered as significantly differentially abundant between breeds or diets when corrected p-value was lower than 0.05 and diff.btw (i.e., median difference in CLR values between factor groups) was higher than 1 or lower than -1. Visualization of coincident DA OTUs between contrasts and pipelines was carried out with VennDiagram package in R [36].

### Pairwise log-ratios analysis

Closured data were also used for another approach, using pairwise log-ratios (p-LR) instead of centred log-ratios [25], as a way to evaluate individual relationships among OTUs and features glommed to genus, family and phylum levels. Recursive Partitioning and Regression Trees (RPART) were generated, including the microorganism pairwise log-ratios most suitable to split samples according to the breed and diet phenotypic criteria.

Pairwise log-ratios analysis is computationally expensive, as feature combinations produce matrices much larger than the original, i.e., a *m* × *n* matrix (*samples* × *features*) would be transformed in a *m* × *n*(*n* − 1)/2 matrix. For this reason, OTU-grouped dataset was size-reduced by selecting a relevant feature subcomposition generated by sparse Partial Least Square Discriminant Analysis (sPLS-DA) (mixOmics package), using the 4-leveled mixed trait “breed-diet” as classification factor. Five-fold cross-validation with permutation was used to test the optimal component structure for the model. In order to generate the subcomposition, OTUs with a Variable Influence of Projection (VIP) value higher than 1 for each component were selected. As p-LR analysis keeps subcompositional coherence [25], data subsetting has no significant effect on the results.

In order to check which microbes were mainly responsible of separation between groups at each ratio, supplementary differential abundance analyses were made using ALDEx2 (as this pipeline uses the same methods as for RPART trees building) with phylum, family and genus-grouped database.

### Microbiome-phenotype association

CLR data were also used to detect associations between microbiome and several phenotypic traits. IMF content was measured in *Biceps femoris* and *Longissimus dorsi*. FA profile was analysed in backfat outer layer (Back) and in ham outer fat layer (Ham). Total FA fractions (saturated, SFA; mono-unsaturated, MUFA; and poly-unsaturated, PUFA) as well as relevant FA contents (oleic, C18:1(9); linoleic, C18:2; palmitic, C16:0; and stearic, C18:0) were also measured, with all quantification methods being described in formerly published studies [11].

PERMANOVA controlling both breed and diet effects was performed for each phenotypic variable using vegan::adonis2 function, in order to elucidate linear or nonlinear relationships between samples distribution and each phenotypic trait:

$$D_{ij}=\mu +b_{i}+d_{j}+P+e_{ij}$$

The additive model applied was built sequentially adding breed (*b*_*i*_; *i = [1*,*2]*) and diet (*d*_*j*_; *j = [1*,*2]*) factors, and each phenotype (*P*) was treated as linear regression at the end. The Aitchison distance matrix (*D*) was used as dependent variable.

Additionally, individual OTU association was addressed using Limma linear regression. To avoid the influence of the known breed effects on phenotype which could lead to potential spurious associations, both microbiome and phenotypic datasets were split for carrying out independent DU and IB microbiome-phenotype analyses. To measure diet weight in correlations, two methods were carried out: (1) linear regression of each OTU on each phenotypic variable without diet correction; (2) linear regression of each OTU on the diet regression residuals of each phenotypic variable. P-values were adjusted using Benjamini-Hochberg method (adjusted significance threshold set to FDR = 0.05). Representation of associations between OTUs and phenotypes was addressed through interaction network in Cytoscape software (v. 3.8.1), setting Kamada-Kawai algorithm (Edge-weighted spring embedded layout) [37] for optimal visualization.

## Results

### Overall microbiota composition

**Fig 1:**
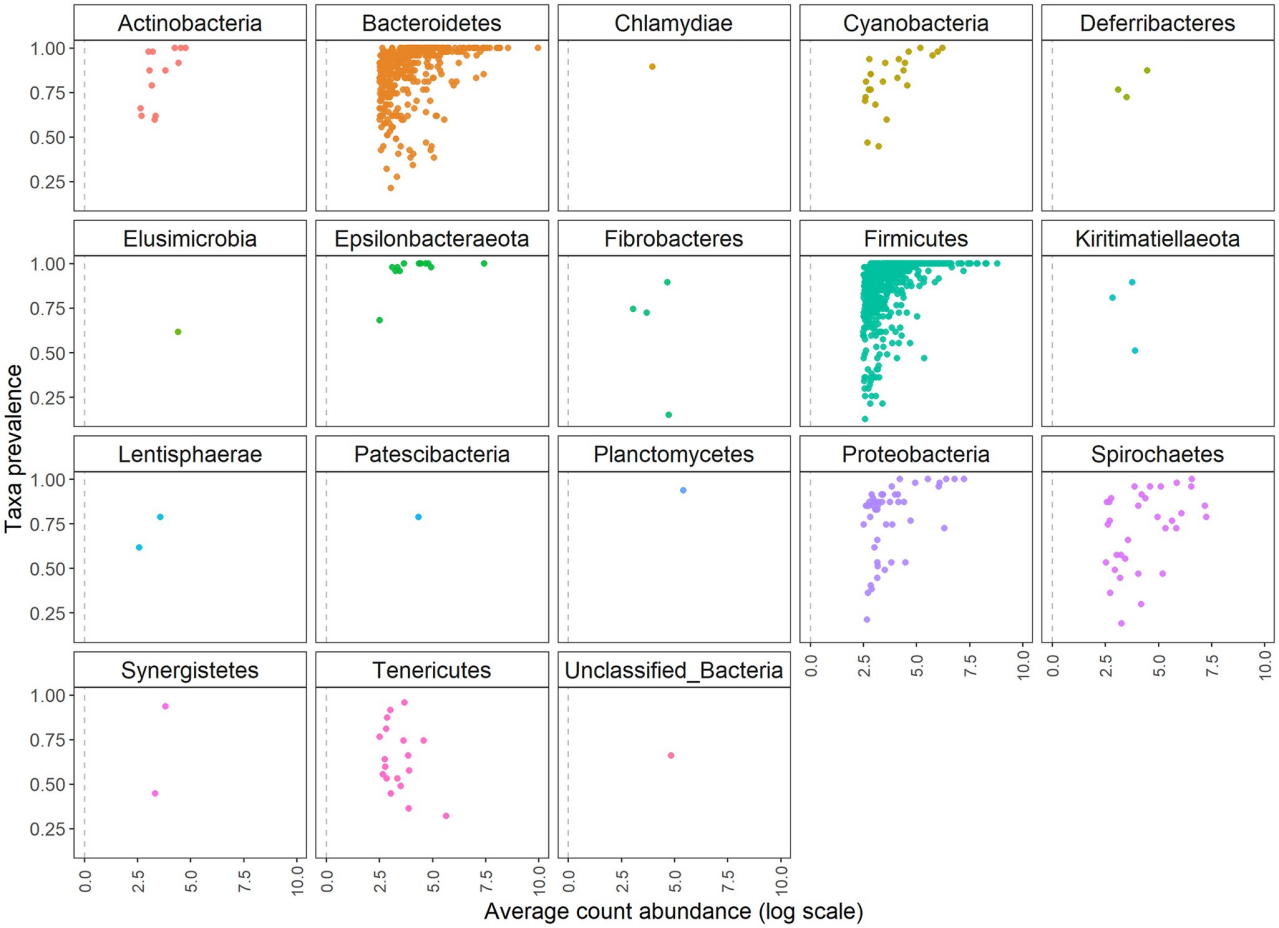
Phyla prevalence and abundance in dataset. Each dot represents an OTU, being the x-axis its average relative abundance and the y-axis the proportion of samples in which it is present. “Unclassified_Bacteria” is the denomination given to those OTUs with no phylum assignment.

The overall composition is summarised in Table 2. Our final OTU table was composed by 47 samples, as we pruned one sample with less than 30k reads. As mentioned in methods, we applied an additional filter removing those reads not assigned to Bacteria kingdom. After this step, 97.44% of total reads were kept, representing 1,246 OTUs. From this bacteria-classified fraction, 99.93% reads were assigned to phylum level. A total of 17 phyla were identified. Prevalence and abundance of phyla are represented in Fig 1.

**Table 2:** Overall microbiota composition.

	Kingdom	Phylum	Class	Order	Family	Genus
% Sequences ^1^	97.44	99.93	99.91	99.91	97.30	86.70
Classified OTUs	1246	1245	1243	1243	1184	988
Identified taxa	1	17	23	33	48	148

Total OTU number, % of sequences assigned to a known taxon at different ranks and unique taxa found at each rank.

^**1**^ Proportion of total reads classified to phylum and lower taxonomic levels are calculated relative to the total reads after filtering OTUs not assigned to Bacteria kingdom.

The 988 annotated OTUs were classified to 148 different genera, covering 86.7% of total reads. 20 genera had an average relative abundance (**RA**) higher than 1%, being *Prevotella_9*, *Lactobacillus*, *Prevotellaceae NK3B31 group*, *Alloprevotella* and *Treponema_2* the most abundant ones (S2 File). Note that SILVA database subdivides some genera according to sequence clustering, appearing under multiple designations in our database.

On the other hand, 79 genera were present at an average abundance lower than 0.1% (S2 File). This fact might be taken into account when interpreting the results, as they might be poorly represented across the samples (i.e., low prevalence) and/or have a minimum number of reads per sample (i.e., low coverage). Nonetheless, all the analyses have been performed including these low-abundance OTUs.

### Microbiota composition analysis

**Fig 2:**
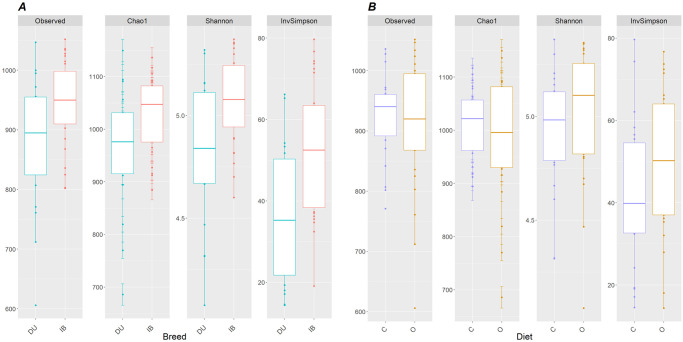
α-diversity boxplots. Alpha-diversity boxplots according to breed (left) and diet (right) groups in rarefied data.

Two-way ANOVA for alpha-diversity measures in rarefied data showed no significant differences between diet groups (C vs O), but all of them (richness: p = 0.021; Chao1 index: p = 0.018; Shannon index: p = 0.008; and Inverse Simpson index: p = 0.002) were significantly affected by breed, these measures showing higher values in the IB group (Fig 2). No significant interaction between breed and diet was detected in the ANOVA. Initial average number of OTUs per sample was 1,046 for DU population and 1,097 for IB population. After rarefaction, these numbers were corrected to richness values, with an average of 880 in DU and 944 in IB.

**Fig 3:**
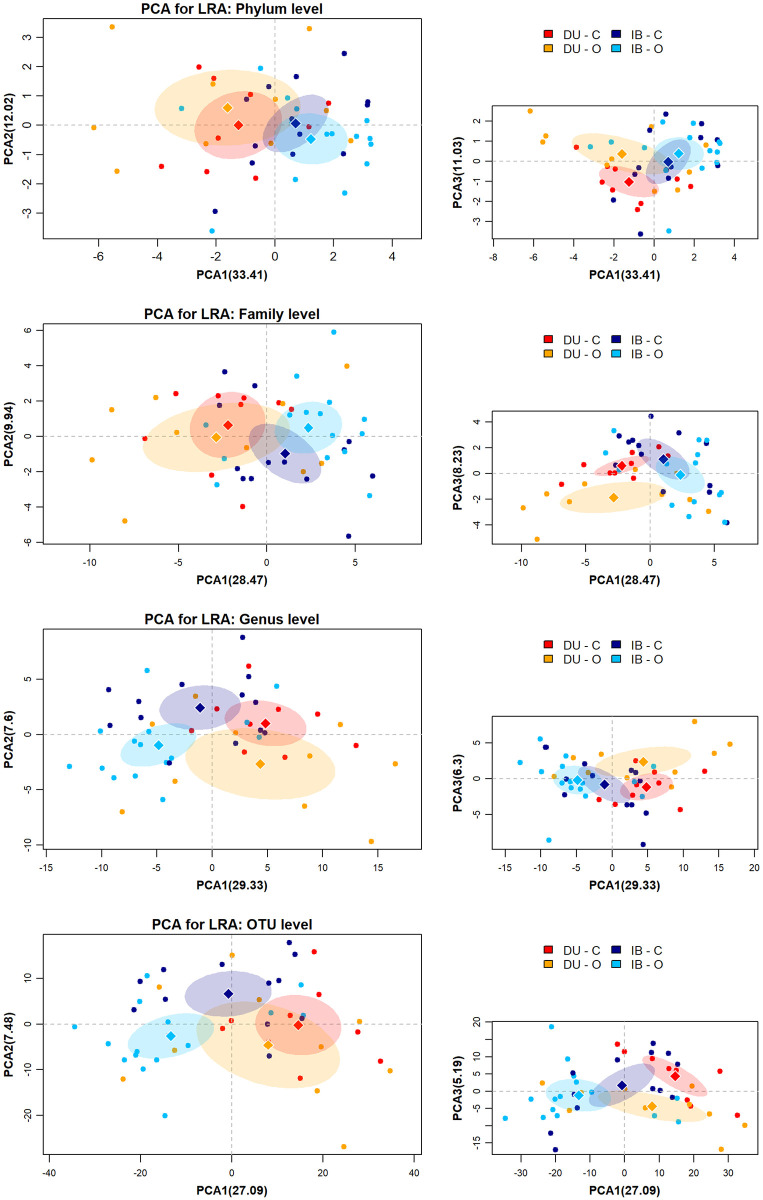
β-diversity plots. Beta-diversity plots from PCA of CLR-transformed data, at phylum, family, genus and OTU levels and variance percentage explained by each component. Ellipses show the Normal-theory confidence regions with alpha = 0.95. Centroids are represented by diamond-shaped points.

**Fig 4:**
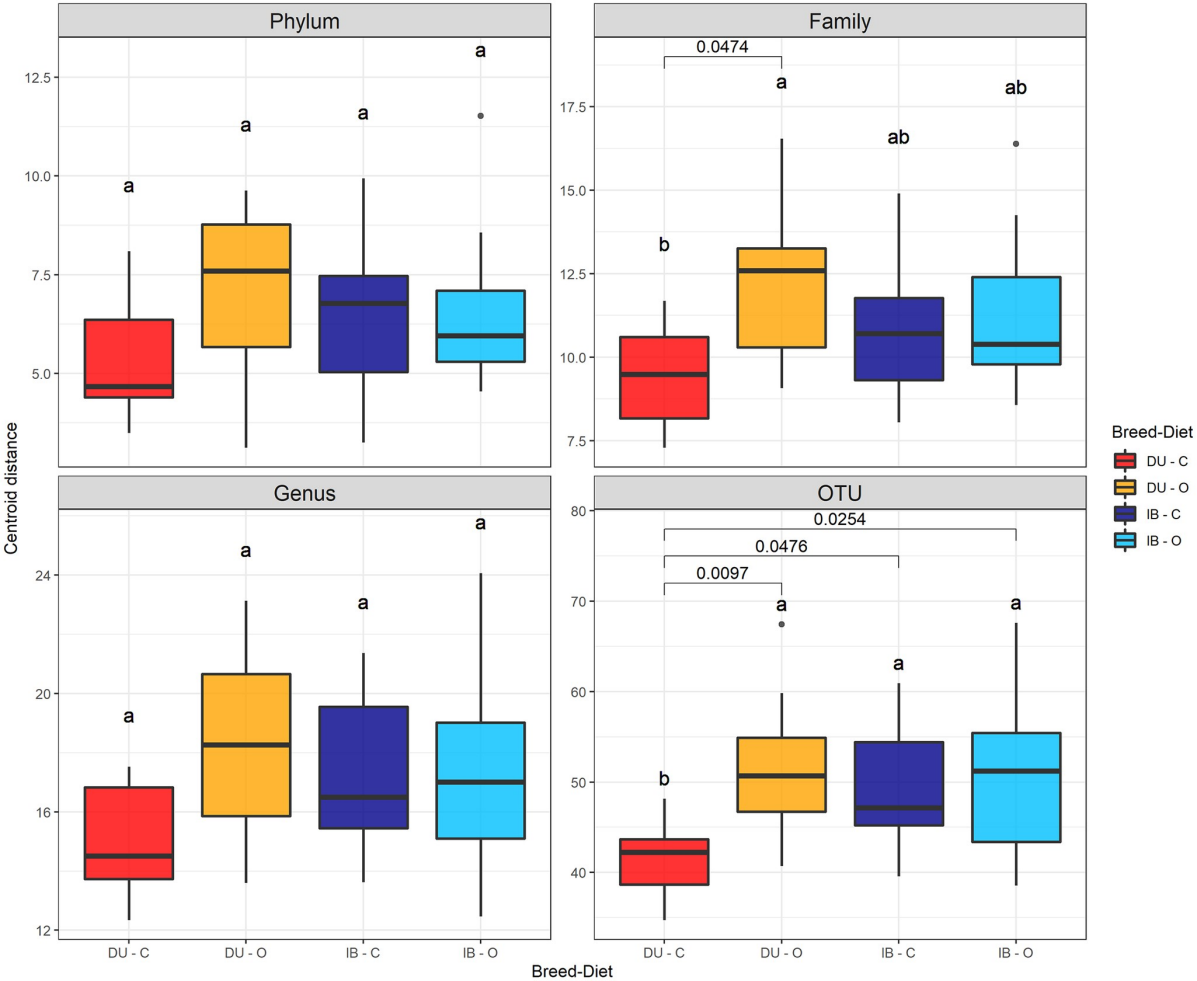
β-diversity indices. Beta-diversity indices by group at different feature glom levels. Tukey HSD test group letters and p-values for significant group comparisons (p < 0.05) are represented.

Compositional PCA for beta-diversity (Fig 3) showed a high dispersion of the data, although breed clustering was observed through PC1. Diet clustering can be observed through PC2, but only when features were analysed at lower taxonomic levels (i.e., genus and OTU levels). Centroid position also revealed that separation between breeds was higher than separation between diets. At OTU level, centroid distance is maximum for both breed and diet groups. Individual beta-diversities were calculated as the Euclidean distance to centroids for groups from mixed “breed-diet” effect. Average individual dispersion within groups was not significantly different between groups, except for family and OTU-glommed datasets. At OTU level, DU-C samples had an average beta-diversity index significantly lower compared to DU-O, IB-C and IB-O groups, although p-value in DU-C vs IB-C contrast (same diet in different breeds) was close to 0.05. (Fig 4). At family level, DU-C individual beta-diversities were also significantly lower than DU-O ones (p = 0.047).

PERMANOVA analysis confirmed that samples from different breeds are significantly different. Differences between diets (corrected by breed effect) are also significant at OTU, genus and family level, although these differences blur at phylum level (Table 3). Nonetheless, the differences between groups are moderate, as 10–12% of variance from our data can be explained by breed effect, and only 3–7% by diet effect.

**Table 3:** PERMANOVA statistics.

		F statistic	R^2^	p-value
**Phylum**	*Breed*	5.05	0.10	0.0017*
	*Diet*	1.44	0.03	0.1776
	*Interaction*	1.32	0.03	0.2187
**Family**	*Breed*	5.96	0.11	0.0001*
	*Diet*	2.24	0.04	0.0283*
	*Interaction*	1.51	0.03	0.1247
**Genus**	*Breed*	6.15	0.12	0.0001*
	*Diet*	2.55	0.05	0.0118*
	*Interaction*	1.27	0.02	0.1859
**OTU**	*Breed*	5.39	0.10	0.0001*
	*Diet*	3.43	0.07	0.0021*
	*Interaction*	1.17	0.02	0.2313

F statistic, R^2^ and p-values at different taxonomic levels.

* p < 0.05.

### Differential abundance (DA) analysis

Two separate contrasts were made, for breed and diet factors, using an FDR cut-off of 0.05 and a fold-change minimum threshold of ±1.5 (*log*_2_ *FC* ≈ ±0.59) for DESeq2 analysis, and a minimum median difference between CLR values of ±1 (*diff*.*btw* = ±1) for ALDEx2 analysis.

**Fig 5:**
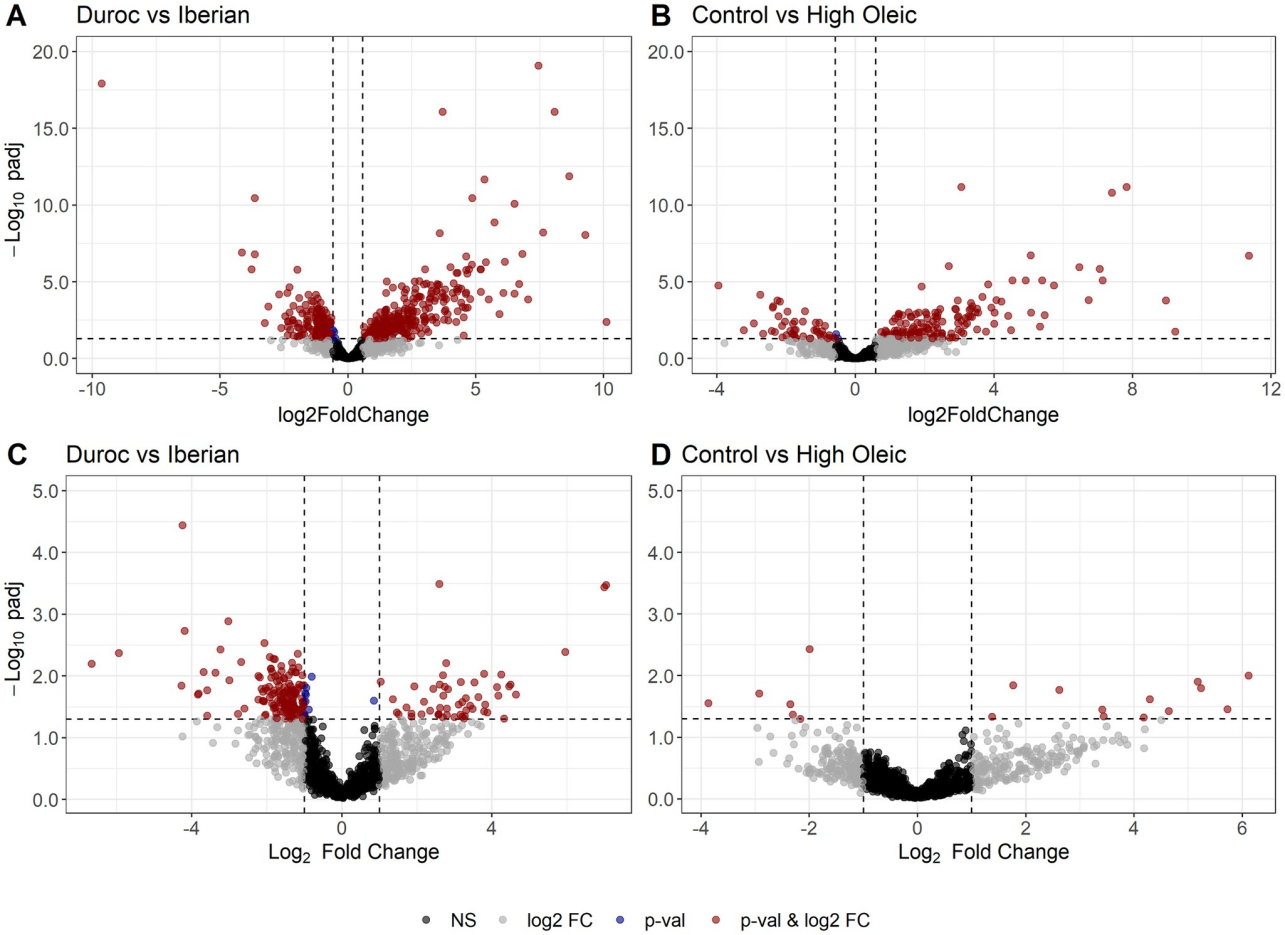
DA volcano plots. Volcano plots of differentially abundant OTUs using DESeq2 (A, B) and ALDEx2 (C, D), for breed (left) and diet (right) contrasts. Red dots represent OTUs with an adjusted p-value below the FDR cut-off (0.05) and a FC value above 1.5 or below -1.5 for DESeq2 contrasts, or a median difference between CLR values above 1 or below -1 for ALDEx2 contrasts. OTUs with Log2(FC) < 0 or diff.btw < 0 are more abundant in Duroc and Control groups, respectively.

Fig 5 includes volcano plots for both contrasts with DESeq2 and ALDEx2 methods. Both breed and diet contrasts resulted in more restrictive results with ALDEx2 method. Breed contrast shows a higher number of over-abundant (**OA**) OTUs in IB pigs when done with DESeq2, while ALDEx2 breed contrast shows a higher number of DU OA OTUs. On the other hand, O diet presents a higher number of OA OTUs than under-abundant (**UA**) OTUs, compared to C diet, both with DESeq2 and ALDEx2. With DESeq2 pipeline, 468 DA OTUs from 119 different genera were found for DU vs IB contrast, mostly from *Ruminococcaceae*, *Prevotellaceae* and *Lachnospiraceae* families. On the other hand, 185 DA OTUs from 71 genera were found between C and O diet groups, predominated by the same families. With ALDEx2, 207 DA OTUs were found for breed contrast, whereas only 18 DA OTUs were found for diet test, mostly from *Prevotellaceae* family. Common DA OTUs between pipelines are counted in Table 4. For breed contrast, the most representative taxa in overlapping DA OTUs was *Prevotellaceae* (mostly *Prevotella*) (27%), followed by *Lachnospiraceae* (16%), *Ruminococcaceae* (15%) and *Veillonellaceae* (mostly *Megasphaera* and *Anaerovibrio* OTUs) (11%). For diet contrast only 18 OTUs were common to both pipelines: 12 were high-oleic OA OTUs from *Prevotellaceae* (7 of them), *Lachnospiraceae*, *Muribaculaceae* and *Ruminococcaceae* families, while the remaining 6 high-oleic UA OTUs were 3 Rikenellaceae members and 3 OTUs classified as Clostridium s.s. 6, Erysipelotrichaceae UCG-004 and Desulfovibrio, respectively. When exploring DESeq2 results, most of the high-oleic OA OTUs were from the same families, but a high number of OA *Prevotella* and *Alloprevotella* OTUs was detected. Within high-oleic UA OTUs, a higher variety of families was present, with high representativity of *Prevotellaceae*, *Ruminococcaceae*, *Rikenellaceae* and *Erysipelotrichaceae*. Supplementary material with the complete DA-OTU list for all contrasts is also available (S3 File).

**Table 4:** DA OTUs by factor and pipeline.

		DA OTUs	DA genera	DA families	DA phyla
**DESeq2**	**Duroc vs Iberian**	468	119	44	13
	**Control vs High Oleic**	185	71	32	9
**ALDEx2**	**Duroc vs Iberian**	207	72	22	6
	**Control vs High Oleic**	18	13	8	3
**Overlap**	**Duroc vs Iberian**	151	63	20	6
	**Control vs High Oleic**	18	13	8	3

Number of DA OTUs in breed and diet contrasts using DESeq2 or ALDEx2 pipelines, and common DA OTUs between both pipelines. Number of genera, families and phyla grouping these OTUs are also shown.

We evaluated coincident DA OTUs among contrasts and methods, as shown in the Venn diagram (S1 Fig). When comparing DESeq2 with ALDEx2, 151 DA OTUs were coincident in the breed contrast and 18 coincidences were found in the diet contrast. On the other hand, when comparing the results obtained for breed and diet effects within the same method, we detected some coincident OTUs (75 OTUs in DESeq2 and only 1 OTU in ALDEx2). Only one OTU is common to all four comparisons, the one named as *denovo378129*, from genus *Shuttleworthia*, which was OA in DU and O groups.

**Fig 6:**
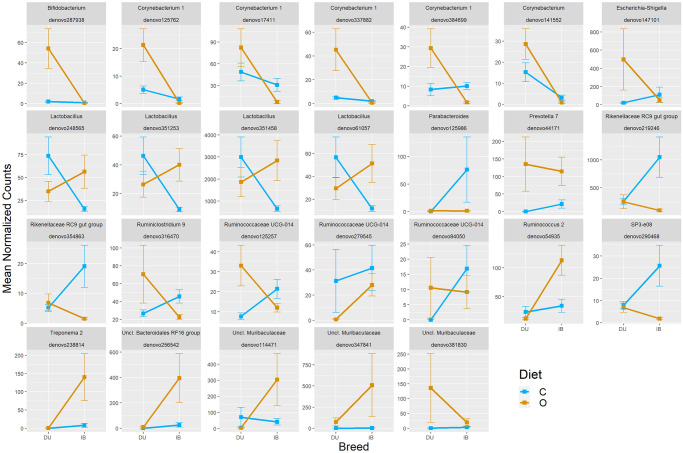
DA interaction plots (DESeq2). Interaction plots for each OTU with significant interaction between breed and diet factors for DA analysis with DESeq2. Normalized counts from DESeq2 algorithm and standard error of the mean (SEM) are represented in y-axis.

Significant interaction between breed and diet was detected for 26 OTUs (P_adj_ < 0.05), most of them being qualitative interactions (Fig 6). Most of these OTUs were members of *Corynebacterium*, *Lactobacillus* and *Ruminococcaceae* taxa.

### Pairwise log-ratio analysis

**Fig 7:**
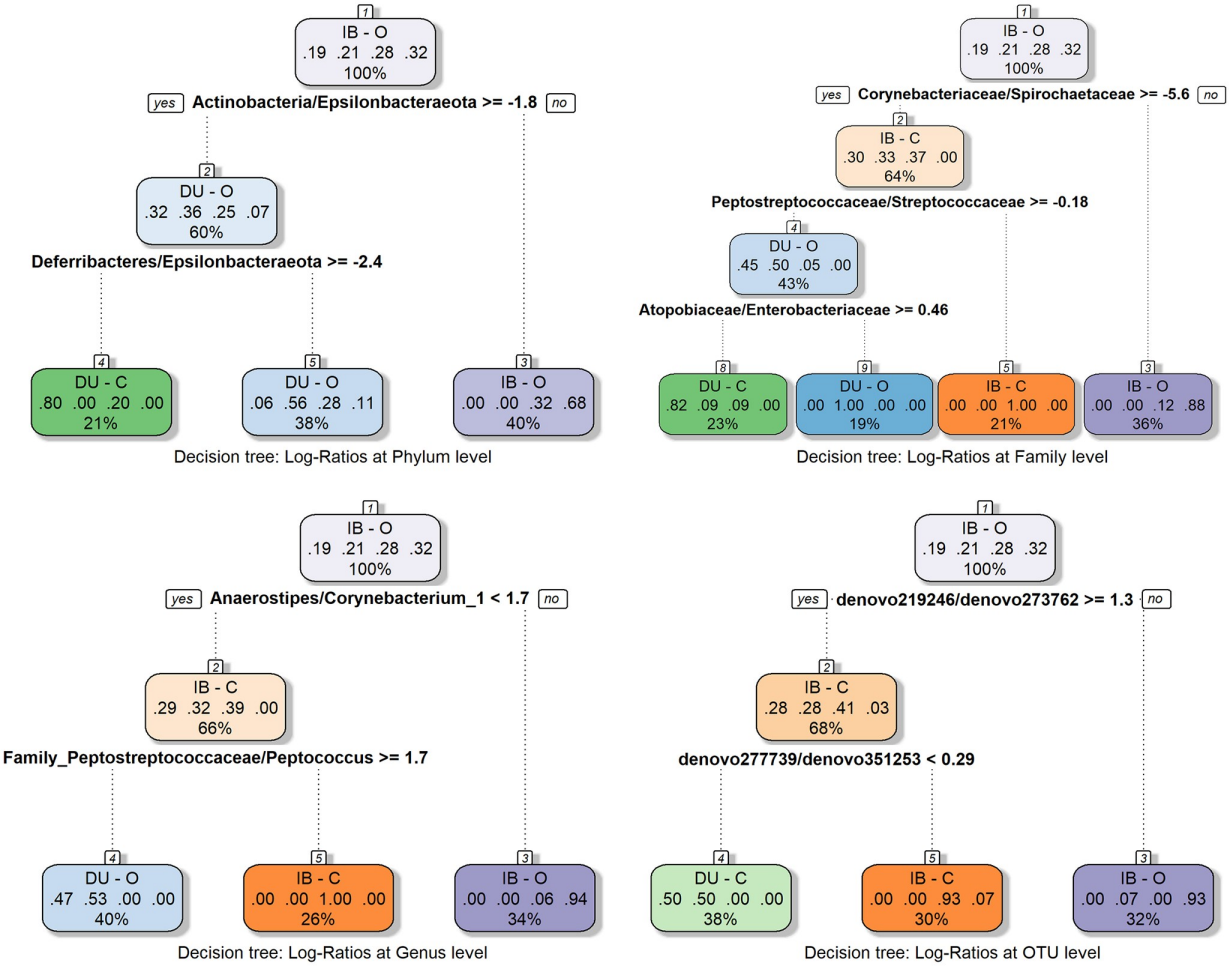
p-LR RPART trees. RPART trees from pairwise log-ratios at phylum, family, genus and OTU levels. Each decision level indicates the threshold value of one p-LR to classify a sample in one phenotypic group or another. Each node box displays the classification, the probability of each class at that node (i.e. the probability of the class conditioned on the node) and the percentage of observations used at that node. Class probabilities at each node are sorted as: DU-C, DU-O, IB-C and IB-O.

sPLS-DA for OTU-grouped dataset confirmed a total of 141 relevant OTUs (VIP ≥ 1) as predictive of the “breed-diet” phenotype. This subcomposition has been used to calculate pairwise log-ratios at OTU level. For genus, family and phylum grouped datasets, full table has been used for p-LR calculation. Recursive Partitioning and Regression Trees (**RPART**) were built for each p-LR matrix, as shown in Fig 7. We observed that the best classification occurs at family level, being the ratios *Corynebacteriaceae*/*Spirochaetaceae* (**COR/SPR**) and *Peptostreptococcaceae*/*Streptococcaceae* (**PEP/STR**) the most relevant to classify between breeds.

At phylum level diet differences were not clear inside each breed, as it was observed in beta-diversity analysis. *Actinobacteria*/*Epsilonbacteraeota* ratio was able to separate samples by breed, being reliable to classify as IB but not as DU, and was not capable of properly separate diet groups inside each breed.

At family level, three ratios were capable of separating animals in the four”breed-diet” groups with high precision. As mentioned, COR/SPR and PEP/STR ratios mainly clustered Iberian and Duroc separately, but also differentiated dietary groups inside the IB breed, while *Atopobiaceae*/*Enterobacteriaceae* (**ATO/ENT**) ratio separated diets within the DU animals. These ratios are closely related with those responsible of group classification at genus level (*Anaerostipes/Corynebacterium_1* and *Family_Peptostreptococcaceae*/*Peptococcus*), except that these genera ratios can only discriminate between IB-C, IB-O and DU animals, but no ratio was able to differentiate diets within DU breed.

In the case of COR/SPR, both ratio components were responsible of variation. ALDEx2 DA analysis at family level showed that both *Corynebacteriaceae* and *Spirochaetaceae* were significantly DA between DU and IB groups (IB-UA: diff.btw = -3.23 and IB-OA: diff.btw = 1.13, respectively) although *Corynebacteriaceae* reduction in IB is stronger than *Spirochaetaceae* increment. As *Corynebacteriaceae* are *Actinobacteria*, breed separation at phylum level is reflected at lower taxonomic levels. Same occurs at the genus decision tree, whose first ratio *Anaerostipes/Corynebacterium_1* was able to differentiate breeds, with *Corynebacterium* having the highest weight, as DA analysis at genus level stated a *Corynebacterium_1* OA in DU animals genus (diff.btw = -2.78).

The PEP/STR ratio was able to identify IB animals, even separating C and O populations when accounting also COR/SPR ratio. DA analysis revealed that *Peptostreptococcaceae* family was more abundant in DU animals (diff.btw = -1.27), while *Streptococcaceae* family was not differentially abundant between breeds. At genus level, *Peptococcus* abundance played a similar role, as IB pigs had a higher RA (diff.btw = 1.14).

As indicated, ATO/ENT ratio was able to distinguish between C and O diets within DU animals, but only when applied after discriminating IB animals with former ratios (otherwise it would only discriminate between DU-O and the rest of”breed-diet” groups). *Enterobacteriaceae* were OA in DU animals (diff.btw = -1.92) but no abundance differences were detected between diet groups.

Finally, the two representative ratios at OTU level (*denovo219246*/*denovo273762* and *denovo277739*/*denovo351253*) were composed by OTUs assigned to *Rickenellaceae*/ *Ruminococcaceae* families and *Peptococcus*/*Lactobacillus* genera, respectively. Additionally, *denovo219246* and *denovo277739* appeared as DA between IB and DU animals (first one with DESeq2 pipeline and second with both DESeq2 and ALDEx2 pipelines), which gave them a substantial weight in breed differentiation.

### Association with phenotypic traits

**Fig 8:**
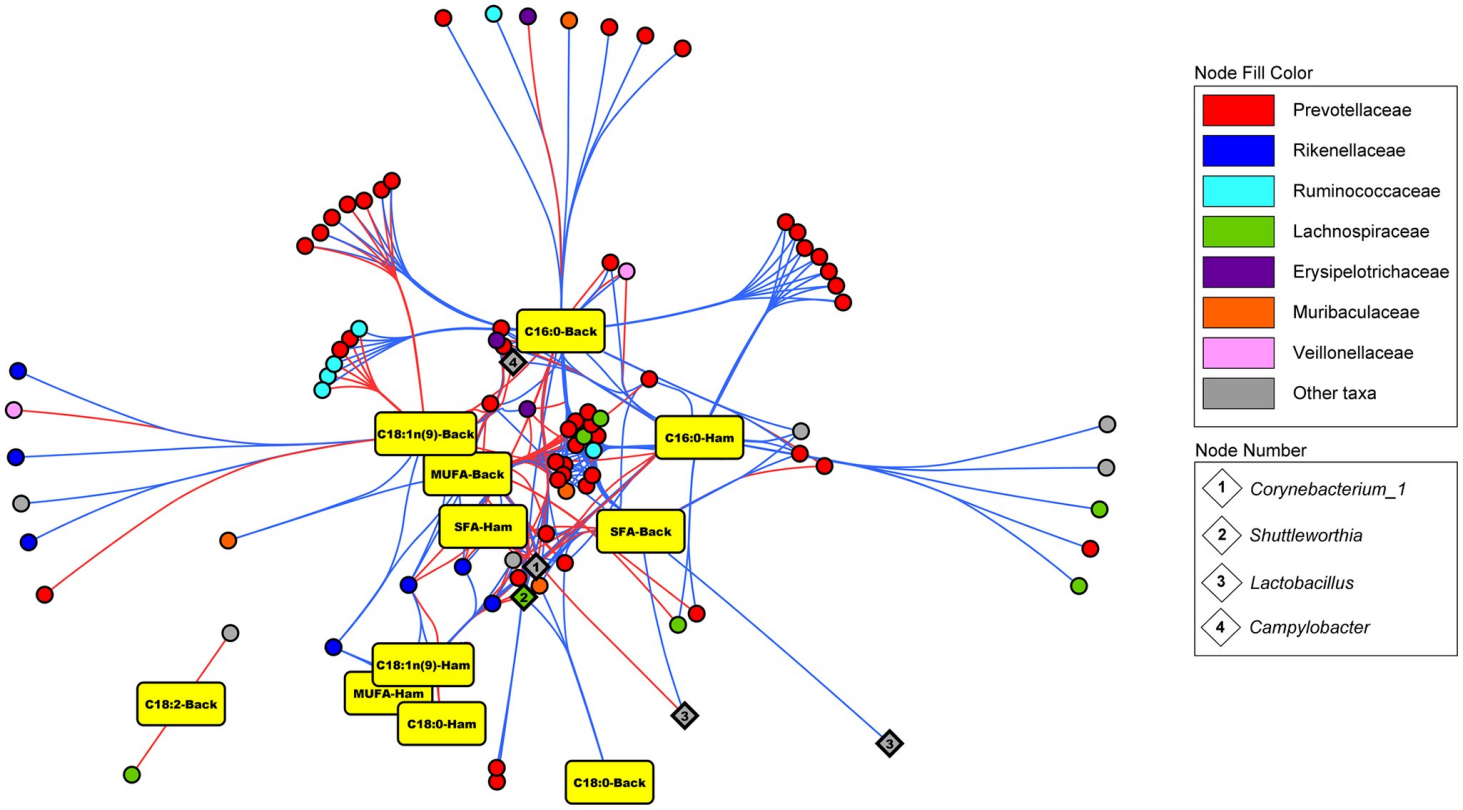
IB OTU-phenotype correlation network. Network representing individual OTU significant correlation with each phenotypic trait within IB subpopulation, from Limma regression analysis, when no diet correction was applied. Edge colour: red = positive correlation; blue = negative correlation. Central nodes have more connections (i.e., significantly associated to more phenotypic traits) than peripheral nodes. Numbered nodes represent OTUs classified as genera with high relevance due to their abundance patterns in our dataset.

PERMANOVA did not show any significant association between overall microbiota composition and the analysed phenotypic traits. When the effect of each individual OTU was evaluated through Limma regression different results were observed for each tested model. Without diet correction, only one OTU was significantly associated to lipidic traits in DU subset, *denovo283234* (*Treponema 2*), which was negatively associated to backfat MUFA and oleic acid content, while, a total of 87 individual OTUs were found as significantly associated to phenotypic traits in IB subset (Fig 8). Most of them belonged to *Alloprevotella* and other *Prevotella* groups and were associated to multiple traits. *Ruminococcaceae* OTUs were positively linked to oleic acid and negatively linked to palmitic acid proportions in backfat, while *Rikenellaceae* OTUs mainly appear negatively associated to oleic acid proportion, affecting in both cases overall MUFA and SFA proportions. Some relevant OTUs from *Shuttleworthia*, *Lactobacillus* and *Corynebacterium* genera also appeared associated to FA composition. When diet correction was applied almost every correlation disappeared, and only one OTU was negatively correlated to ham oleic acid proportion in IB subset, *denovo37314* (*Treponema 2*).

## Discussion

In this study we explore the composition and disparities of the bacterial fraction of fecal microbiota populations between two different pig breeds (Iberian and Duroc) at 117 days old, fed diets with different energy source (carbohydrates *vs*. sunflower oil with high oleic acid content). Phenotypic characterization of these animals has been previously reported by Benítez *et al*. (2018) [38]. Briefly, breed effects affected feed intake, fatness and premium cuts’ yield with Iberian animals showing higher feed consumption, thicker backfat and lighter hams, with no differences in body weight. Regarding diet, differences were observed in FA composition, as the high-oleic group had higher MUFA and oleic acid, and lower SFA than the control group.

Overall taxonomic structure revealed *Bacteroidetes*, *Firmicutes* and *Proteobacteria* as the most abundant phyla, with an important predominance of *Prevotellaceae*, *Lachnospiraceae* and *Ruminococcaceae* families. *Prevotella* genus was the most abundant, with an average RA of 30% in Duroc animals and 22% in Iberian animals when considering all SILVA genus-clustering designations. *Prevotella* are anaerobic saccharolytic bacteria which produce acetate and succinate through fermentation [39] and their abundance is highly related to high-fiber long-term diets [40, 41]. Their presence in fecal microbiota is highly reported in human [42] and other animals such as ruminants, predominating in rumen microbiota [43–45]. Multiple studies have shown the dominance of *Prevotella* in pig gut microbiota [46] as well as their relevance in enterotypes [47] or even immune response [48]. *Alloprevotella* is also one of the most representative genus in our data, with an average RA of 4.2% in DU and 11.6% in IB animals. This genus is also known for its saccharolytic activity [49], although it is less studied than *Prevotella* due to its lower abundance in gut communities. *Lachnospiraceae* and *Ruminococcaceae* families also comprehend well-known polysaccharide fermenters, which in addition participate in methanogenesis by producing H_2_ and formate as substrates to archaeal communities [50]. Abundance balance of these three families in gut microbiota, through a number of abundance ratios, might be relevant for host traits related with feed efficiency, metabolism or health. For instance, *Prevotella*-to-*Bacteroides* ratio has been related with weight and fat loss in humans [51], and *Firmicutes*-to-*Bacteroidetes* ratio has been repeatedly associated to fiber metabolism or obesity in human populations [40, 52] and murine models [53], although there is controversy about these links [54]. Our data support that this core microbiota might also be shared by other monogastric species along all digestive tract. Similar findings are reported by Crespo-Piazuelo *et al*. [55] when analysing gut microbiota in different intestinal regions using the same IB-C pig population. However, they reported a range of 35 to 41% of *Prevotella* abundance within all colon regions. These discrepancies must be attributed to different intestinal region, different genotype and diet of the total animal population studied (colon from IB-C animals vs rectum from IB and DU fed C and O diets).

The pipeline used for analysing differences between experimental groups included a classic alpha-diversity evaluation and a compositional approach to beta-diversity. Differential abundance analysis was used to acquire a more specific insight about changes in microbiome population structure. DESeq2 is a RNAseq-based, robust and well documented method broadly used in microbiome DA analyses. However, in recent years the concern about compositional nature of these data has increased, and some authors remark that RNAseq-based tools are not accounting for the compositional nature of microbiome data. For this reason, we also performed differential abundance analysis with ALDEx2 method, an ANOVA-based approach which accounts for this intrinsic compositionality and has a similar sensitivity than other methods, also reducing false positive rate near to zero [31]. The most conservative strategy would be to focus in the overlapping DA OTU set between DESeq2 and ALDEx2, but due to the restrictive behaviour of ALDEx2 we decided to consider also DESeq2 results for further exploration. Furthermore, pairwise log-ratio (p-LR) analysis was also used to deepen in composition differences, as ratios between OTUs can also be biologically relevant, beyond single association with OTU RAs. This approach allowed to detect those microorganism pairwise ratios whose abundances could separate animals by both breed and diet.

Our results revealed an important effect of the host genetic background on the fecal microbiota composition, both for bacterial richness and overall composition. Regarding richness, a higher number of different OTUs is observed in IB pigs, which can also explain the higher values of Shannon and Simpson indices calculated for this breed. These interesting findings might be related with the higher rusticity and resilience of Iberian pig breed compared with other commercial breeds [56], since higher ecological diversity is associated with a better response to environmental disturbances (response diversity) [57]. Regarding composition, fecal microbiome resulted more different between breeds than between diets, as PCA sample distribution, PERMANOVA and DA analyses revealed. DU animals have a higher abundance of *Actinobacteria*, specifically from *Corynebacterium* and *Bifidobacterium* genera, as well as multiple *Lachnospiraceae* (*Blautia*, *Roseburia*, *Lachnoclostridium*, *Fusicatenibacter*, and *Shuttleworthia*), multiple *Prevotellaceae* OTUs (mostly *Prevotella* and *Alloprevotella*) and other genera such as *Clostridium s*.*s*. *1*, *Holdemanella*, *Megasphaera* or *Succinivibrio*, while Iberian OA OTUs were scarcer and belonged to more diverse taxa (see S3 File for complete OTU list). As far as we know, no former studies have compared Duroc and Iberian pigs’ microbiota, so these findings are novel and might be important to define unique microbiome patterns associated to host genotypes. Still, gut microbiota contrasts between Duroc, Landrace and Large White pigs also revealed a higher presence of *Prevotella* in Duroc animals [15].

Focusing on Actinobacteria, we specifically found a higher abundance of *Bifidobacterium* and *Corynebacterium* in DU animals. *Bifidobacterium* are well known anaerobic lactic acid producer bacteria [58] whose abundance in fecal microbiota has been reported as negatively correlated with pig age, being more abundant at early life stages [59]. The transition from weaning to adult cereal-based diet is a key factor in the replacement of these early-colonizers by other microorganisms such as *Prevotella*, *Roseburia* or *Succinivibrio*, more typical of adult fecal microbiota [48]. Only one *Bifidobacterium* OTU was present in our data, presenting low abundance (mean RA = 0.01%) and majorly absent in IB animals. This might indicate a slower replacement of weaning to adult microbiota in DU pigs, which might as well be related to their lower alpha-diversity. *Corynebacterium* includes a wide variety of gram-positive aerobic and facultative species, some of them being fermentative organisms with special relevance in amino acid biosynthesis pathways, such as those of lysine or histidine [60, 61]. Lower protein deposition rates have been reported in Iberian pigs, compared to other commercial breeds [62]. In agreement, our Duroc pigs showed higher muscle deposition, as lower fat and higher ham yield was observed with similar body weight respect to Iberian pigs [38]. Thus, *Corynebacterium* might be contributing to a differential protein synthesis and degradation balance, through a differential bioavailability of essential amino acids in the gut, thus, their abundance could be correlated to animal protein deposition.

Differential abundance of other groups might as well be related with richness and resilience of Iberian pigs. In this sense, the lower abundance of *Shigella-Escherichia* in IB pigs might be related with the previously reported increase of microbial diversity, as a higher alpha-diversity has been associated to the prevention in the establishment of pathogenic microbes such as *Enterobacteriaceae*. However, this association has only been described in humans [40] and our data do not confirm this finding. Likewise, the mentioned increment in richness seems to be related with the lower abundance of multiple *Prevotella* OTUs in IB pigs, as it happens when comparing C and O diets. *Treponema* DA OTUs, which are virtually absent in Duroc animals, appear as moderately abundant in Iberian pigs (RA ≈ 1%), thus these fiber-fermenters might have settled the niche that *Prevotella* left. Finally, we detected an abundance opposition between *Prevotella* and *Treponema* taxa: we found a number of *Prevotella* OTUs with high RA being over-abundant in DU animals, while several *Treponema* OTUs were over-abundant in IB animals. As described by Crespo-Piazuelo *et al*. [55], this might suggest a competition for dietary fibre.

The diet effect on microbiome appears masked by the breed influence, but its impact on microbiome structure is also noticeable, since PERMANOVA revealed significant differences in distances between samples from different diets in relation to distances within each diet. It must be pointed out that O diet had a higher fiber content than C diet, as it was formulated to be isoenergetic in relation to C diet. This fact might be related to the greater presence of *Prevotella* and kindred taxa in O animals, since fiber content has already been linked to gut microbiota changes [63] and *Prevotella* OTUs have been defined as biomarkers for high-fiber diets [64]. Generally speaking, high-oleic OA OTUs were rare OTUs (i.e., RA ≤ 0.01% in C group), while control diet OA OTUs tended to be more common (i.e., RA > 0.01% in C group), meaning that the abundance decrease of common OTUs due to oleic acid and fiber supplementation results in an abundance increment of marginal OTUs. This fact is supported by our Shannon index values showing an upward trend in O group compared to C diet, which, in this case, means a higher overall evenness in O group, as observed richness is not different between diets.

Regarding the combined effect of breed and diet, no significant interaction was detected between both factors for alpha and beta-diversity. Nevertheless, PCA showed a slightly different separation of C and O centroids within each breed, which could suggest a different response to diet between Duroc and Iberian microbial communities. Also, within-group dispersions revealed a possible variation in diet response based on breed, as DU-O dispersions tended to be higher than the rest, while DU-C dispersions were the lowest (these differences being significant at OTU level). Conversely, IB-C and IB-O dispersions were similar and intermediate. The higher dispersion level within DU-O cluster compared to DU-C is coincident with the increment in alpha-diversity variability detected for this group, especially when observing richness or Chao1 index values, and opposes to the sample homogeneity within DU-C. In contrast, IB-O animals did not show different alpha-diversity mean or dispersion values compared to IB-C. Thus, it can be hypothesised that Duroc animals might respond in a more heterogeneous way to dietary oleic acid increment.

We found a unique OTU, *denovo378129* (*Shuttleworthia*) as differentially abundant between all breed and diet groups. This bacterial group is relatively unknown, although it has been reported as a typical member of animal gut microbiome in several studies [59, 65]. According to our results, *Shuttleworthia* could be a key differentiator between Iberian and Duroc animals, having a relevant link either with fiber or with oleic acid dietary supply. Additionally, DESeq2 allowed us to test interaction effects between breed and diet factors. With this method we were able to find as much as 26 significant interactions, most of them being qualitative. According to DESeq2 DA analysis for diets (C vs O) using DU and IB subpopulations, we were able to classify these interactions based on the different response to diets inside each breed: no response to O diet (NR), abundance increase due to O diet (AI) or abundance decrease due to O diet (AD). 12 OTUs were NR within DU animals (7 IB-AI and 5 IB-AD) while 7 OTUs were NR within IB animals (all of them DU-AI). Only 1 OTU (*denovo347841*) presented an abundance increase in response to O diet within both breeds, being its AI much larger for Iberian animals (quantitative interaction). The remaining 6 interaction-significant OTUs were not significantly DA between diets within each breed. The OTU *denovo347841* has been classified as *Unclassified_Muribaculaceae*, a relatively unknown *Bacteroidales* family which has been observed in multiple gut microbiomes through 16S or metagenomic analyses [66]. Although their main metabolic role remains unknown, their phylogenetic closeness to *Prevotellaceae* and *Bacteroidaceae* could reveal saccharolytic activity. In summary, most of the observed interactions are qualitative and reveal that one breed responds to diet but the other does not. No pure qualitative interactions (i.e., with opposite directions of diet effect according to breed) were observed. The presence of interactions for individual OTU abundances would be a clear indicator of differential effects of diet on the gut microbiota of Duroc and Iberian pigs. This fact can be related with our beta-diversity results, as our PCA showed a tendency to a higher centroid distance within IB-C and IB-O groups than within DU-C and DU-O, although the absence of significant interactions in PERMANOVA test throws some uncertainty about this conclusion. Also, former studies carried out with the same animals have proven the existence of a differential response to O diet for several productive traits and for adipose tissue transcriptome [67]. In summary, this differential response may exist for gut microbiome composition and structure, but might be not clearly noticed due to the low number of animals and to the relatively high resemblance between feed formulations and to the intrinsic difficulty in evaluating and interpreting interaction effects.

The p-LR approach must be mentioned apart, as it identifies ratios that could be useful as differentiators between breed and diet combinations, although distinction between Duroc and Iberian phenotypes is clearer. *Actinobacteria* abundance has been observed to be important when compared to *Epsilonbacteraeota* (Phyl. Nov.), a novel phylum proposed by Waite *et al*. [68] as a separate group from former *Epsilonproteobacteria* class, which was classified inside *Proteobacteria* phylum. Our dataset contained *Campylobacter* and *Helicobacter* members of *Epsilonbacteraeota*, two genera with diverse metabolisms more known by their pathogenic species affecting human and other animals. One out of our OTUs has been classified as *Campylobacter hyointestinalis*, a species name proposed to *Campylobacter* isolated from pigs with proliferative enteritis [67]. Interaction relationship of these pathogenic bacteria and *Actinobacteria* (*Corynebacterium*, *Bifidobacterium*, *Collinsella*, *Enterorhabdus* and non-classified *Atopobiaceae*) present in our database remains unknown. However, as *Epsilonbacteraeota* RAs remain fairly constant across samples, differentiation between IB and DU pigs might be due to lower *Actinobacteria* abundances in Iberian animals. Actinobacteria relevance in breed separation has been upheld when analysing ratios at lower taxonomic levels. Accordingly, *Corynebacteriaceae*/*Spirochaetaceae* and *Anaerostipes*/*Corynebacterium* were relevant in breed separation at family and genus levels, respectively. In any case, complete separation between breeds and diets, including the discrimination between Duroc diet groups, is only possible at family level making use of three pairwise ratios (COR/SPR, PEP/STR and ATO/ ENT), considering that *Enterobacteriaceae* are more abundant in DU-O group. This fact also reflects the masking effect that breed is causing to differentiate diet effects on microbial populations, previously commented.

This type of approach might be interesting to detect microbial biomarkers, given their importance as tools for genetic diagnosis or diet determination. In the case of Iberian pig industry this might be especially useful in order to address frauds due to crossbreeding and dietary manipulation, which can importantly affect final products’ quality [6, 7]. Nevertheless, additional studies including crossbred animals, different production systems and sampling and different developmental stages, including the standard slaughter age, are necessary in order to validate these potential biomarkers and establish causal relationships.

As an additional approach we related the individual OTU abundances with different fat composition traits, in order to deepen in the potential effects of the gut bacteria on host’s metabolism. A former study with the same animals reported significant differences in FA composition between C and O diets [38]. Our first Limma approach (breed subsetting and no diet correction) was used to globally visualize the correlations between OTU abundances and lipidic composition, independently of their causal relationship. Again, differences were detected between DU and IB, as only one OTU abundance was significantly correlated to FA composition in DU animals. When the second Limma approach was applied (breed subsetting and diet correction) we saw that most of these correlations were caused by the diet influence. In IB subpopulation, *Prevotellaceae* OTUs mostly appear negatively correlated to SFA and/or positively correlated to MUFA (as the variation in one FA alters the proportion of the others). As they have a role in fiber digestion, these correlations might be caused by the higher fiber content in high-oleic diet, as mentioned before. Other correlations might be directly caused by increased supply of dietary oleic acid, such as the negative correlations between OTUs from *Lactobacillus* (*denovo2335*, *denovo274149*) and backfat SFA content, or between *Campylobacter* (*denovo141328*) and both ham and backfat SFA content. *Lactobacillus* are important fermenters with a crucial role in VFA biosynthesis [69], so its association with FA availability could be expected. Regarding *Campylobacter*, as it happens with *C*. *jejuni*, *C*. *hyointestinalis* might also lack common microbial metabolic pathways related to carbohydrate utilization, replacing them by a rapid utilization of amino acids and SCFA such as lactate and acetate [70]. Thus, its correlation with FA composition might be related to differential SCFA and amino acid bioavailability between dietary groups. Only one OTU was detected as correlated with FA after correcting by diet (second Limma approach), *denovo37314* (*Treponema 2*), negatively correlated with ham oleic acid content in IB. This suggests that direct (i.e., not caused by diet differences) association between gut microbiota and FA muscle accretion might also exist. Unfortunately, our experiment does not allow to detect these associations, as intra-group variability is very low for lipidic traits and sample size does not provide enough statistical power. Further experimental designs standardizing breed and diet might be adequate to focus on causal relationships between gut microbiota composition and host lipidic traits.

As our experiment has focused on the bacterial subset of fecal microbiota, interactions with other microbial clades, especially eukaryotes, must be studied for a better understanding of the real nature of microbial population changes. On the other hand, metabolite variations between breeds and diets must be analyzed in order to stablish the true relationship between microbiome and host phenotype. Mid-gut microbiota populations might also be interesting to observe dietary responses, as multiple digestion processes occur at superior gut sections. Finally, the complexity in analysing crossover designs must be taken into account, and additional experiments focused in a unique effect might help to clarify some of the differences found in this study, although interaction between effects must not be ignored.

## Conclusions

Our study reveals that genetic background has an important impact on pig gut microbiota composition, while dietary changes have a smaller and more variable effect, and it also brings light to the complexity of microbial relationships. In this experiment, the O diet provided a high level of oleic acid content instead of carbohydrates as energy source, but also higher fiber content was mandatory to keep both diets isoenergetic, thus the effects of diet on microbiota composition could be highly dependent on fiber. This work is the first comparison of breed and diet effects on gut microbiome involving Iberian pigs, providing novel and interesting results, but limitations due to the sample size must be taken into account. We report a higher OTU richness in Iberian pigs, which highlights the importance of genetic background (i.e., animal breed) in overall microbiome composition resemblance, and may suggest a potential relationship between gut microbiota diversity and composition and Iberian pigs’ resilience. Dietary modifications had a small effect in microbiome composition, much less important than host genotype. Differential abundance revealed a complex picture of microbe relationships, with a considerable effect of the breed and multiple interactions between breed and diet. In spite of this complexity, we identified relevant DA taxa and taxa ratios as potentially associated to the metabolic differences between breeds and to a lower extent for the diet influence. Finally, an approach to the use of OTU pairwise ratios as a predictive tool has been performed, with some interesting results that should be subject of further investigations.

## Supporting information

S1 Fig DA Venn diagrams. Venn Diagram comparing DA OTUs in breed (red) and diet (blue) contrasts using both DESeq2 and ALDEx2.
(TIF)

S1 File Diet composition. Nutrients, fatty acid composition and ingredient formulation for Control (C) and High-Oleic (O) diets.
(PDF)

S2 File Relative abundance of genera. Tables containing overall relative abundance of identified genera and relative abundance by”breed-diet” groups. Mean and standard deviation of RA are shown for each OTU, as well as taxonomic classification. RA_Rank column represents the rareness of each OTU (High_RA: RA ≥ 1%; Med_RA: 1% > RA ≥ 0.1%; Low_RA: RA < 0.1%).
(XLSX)

S3 File Differentially abundant OTUs. Tables containing differentially abundant OTUs for each factor (breed and diet) with both pipelines (DESeq2 and ALDEx2) and overlapping of both pipelines. Prevalence represents the proportion of samples in which each OTU is found. For DESeq2, normalized counts per group (Ncounts), fold change (log2FC) and adjusted p-value (padj) are shown. For ALDEx2, relative abundance per group (RA), median difference in CLR values between factor groups (diff.btw) and Welch’s test adjusted p-value (we.eBH) are shown. In overlapping tables information of both pipelines is shown.
(XLSX)

## Abbreviations

AD: Abundance decrease
AI: Abundance increase
ATO/ENT: *Atopobiaceae/Enterobacteriaceae*
BW: Body weight
CLR: Centred log-ratio
COR/SPR: *Corynebacteriaceae/Spirochaetaceae*
DA: Differential abundance
FA: Fatty acid
FC: Fold-change
FDR: False discovery rate
IMF: Intramuscular fat
MUFA: Mono-unsaturated fatty acids
NR: no response
OA: Over-abundant
OTU: Operational taxonomic unit
PCA: Principal Component Analysis
PEP/STR: *Peptostreptococcaceae/Streptococcaceae*
PERMANOVA: Permutational multivariate analysis of variance
p-LR: Pairwise log-ratio
PUFA: Poly-unsaturated fatty acids
RA: Relative abundance
RPART: Recursive Partitioning and Regression Trees
SCFA: Sort-chained fatty acids
SFA: Saturated fatty acids
sPLS-DA: sparse Partial Least Square Discriminant Analysis
UA: Under-abundant
VIP: Variable Influence of Projection
