# Supplementary figures and images for: Influence of genetic background and dietary oleic acid on gut microbiota composition in Duroc and Iberian pigs
Source: PLoS One. 2021 May 20;16(5):e0251804. doi: 10.1371/journal.pone.0251804 (PMC8136687; doi:10.1371/journal.pone.0251804)

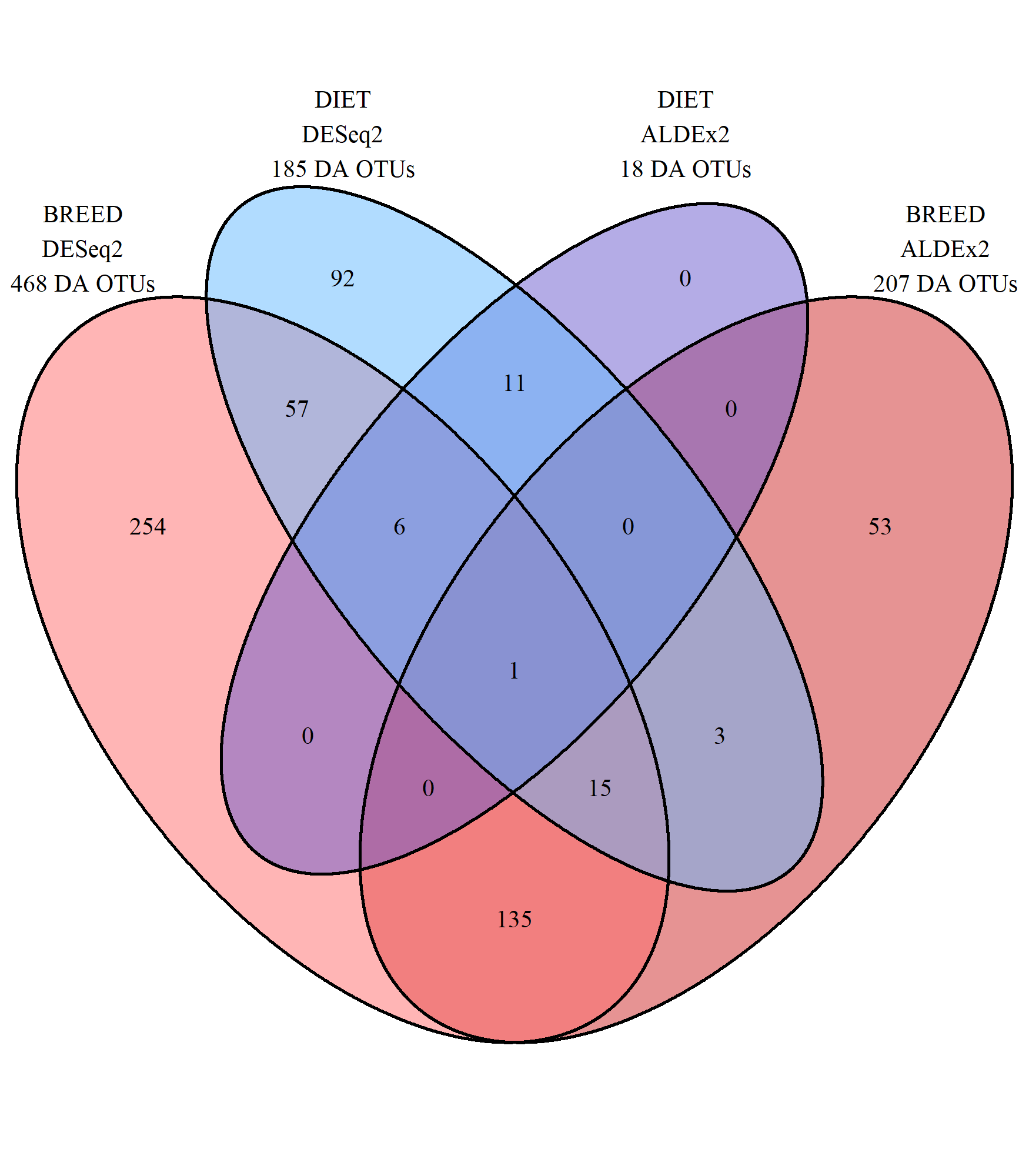

Supplement: S1 Fig — Venn Diagram comparing DA OTUs in breed (red) and diet (blue) contrasts using both DESeq2 and ALDEx2. (TIF) [file pone.0251804.s001.tif]
