# Supplementary material for: Influence of genetic background and dietary oleic acid on gut microbiota composition in Duroc and Iberian pigs
Source: PLoS One. 2021 May 20;16(5):e0251804. doi: 10.1371/journal.pone.0251804 (PMC8136687; doi:10.1371/journal.pone.0251804)
Supplement: S1 File — Nutrients, fatty acid composition and ingredient formulation for Control (C) and High-Oleic (O) diets. (PDF) [file pone.0251804.s002.pdf]

S1 File– Nutrients (1), fatty acid composition (2) and ingredient formulation (3) for Control (C) and High-Oleic (O) diets.

O Diet is enriched with 6% of high-oleic sunflower oil. Both diets were formulated according to FEDNA nutritional guidelines (2006) using the Brill Formulation software (Brill Co., Georgia, USA) to be isocaloric and isoproteic.

1) Nutrient composition	C DIET – 0%		O DIET – 6%	
**Nutrient**	**Value**	**Unit**	**Value**	**Unit**
Dry Matter	87,42	%	88,81	%
Digestible energy (DE)	3.320	kcal	3.318	kcal
Metabolizable energy (ME)	3.230	kcal	3.230	kcal
Net energy (NE)	2.413	kcal	2.458	kcal
Crude Fat	2,453	%	7,765	%
Palmitic A. C16:0	0,272	%	0,436	%
Stearic A. C18:0	0,035	%	0,200	%
Oleic A. C18:1n-9	0,436	%	4,650	%
Linoleic A. C18:2n-6	1,104	%	1,718	%
Starch	48,190	%	36,840	%
Sugars	3,385	%	3,599	%
Crude fiber	2,971	%	4,527	%
Acid detergent lignin (ADL)	0,747	%	1,230	%
Acid detergent fiber (ADF)	3,797	%	5,900	%
Neutral detergent fiber (NDF)	10,990	%	15,288	%
Crude protein	15,600	%	15,600	%
Met	0,250	%	0,239	%
Met + Cys	0,556	%	0,538	%
Lys	0,800	%	0,800	%
Thr	0,571	%	0,564	%
Trp	0,182	%	0,200	%
Digest. Met	0,227	%	0,210	%
Digest. Met + Cys	0,498	%	0,465	%
Digest. Lys	0,700	%	0,678	%
Digest. Thr	0,488	%	0,464	%
Digest. Trp	0,160	%	0,168	%
Crude Ash	4,434	%	6,791	%
Ca	0,750	%	0,750	%
Ca An.	0,650	%	0,650	%
P	0,610	%	0,622	%
P An.	0,510	%	0,522	%
Available P	0,355	%	0,368	%
Digest. P	0,295	%	0,295	%
Na	0,171	%	0,174	%

2) FA composition	C DIET – 0%	O DIET – 6%
**Fatty Acid**	**mg/g**	**mg/g**
C14:0	0.14	0.13
C16:0	4.83	7.19
C16:1n-9	0.02	0.04
C16:1n-7	0.06	0.17
C17:0	0.07	0.08
C18:0	0.84	1.83
C18:1n-9	9.47	36.82
C18:1n-7	0.23	4.43
C18:2n-6	14.24	16.68
C18:3n-3	0.99	1.21
C20:0	0.10	0.22
C20:1n-9	0.12	0.23
C20:3n-3	0.01	0.04
C20:5n-3	0.04	0.11
C22:1n-9	0.01	0.02
C22:4	0.03	0.05
C22:5n-3	0.17	0.04
C22:6n-3	0.16	0.36
**SUM FA**	**31.53**	**69.65**

3) Ingredient formulation

C DIET – 0%			O DIET – 6%		
**Ingredient**	**%**	**kg**	**Ingredient**	**%**	**kg**
MAIZE PB7.7 A63.5	33,91	339,07	COMMON WHEAT PB11 A59.5	25,00	250,00
COMMON WHEAT PB11 A59.5	30,00	300,00	BARLEY PB10 A51.5 FB4.5	38,87	388,75
BARLEY PB10 A51.5 FB4.5	16,87	168,69	WHEAT BRAN FB8.5 PB15	7,00	070,00
SOYBEAN MEAL PB47	16,88	168,78	SOYBEAN MEAL PB47	15,53	155,30
CALCIUM CARBONATE	0,83	008,27	DEHYDRATED ALFALFA PB 16	3,50	035,00
DICALCIUM PHOSPHATE DIHYDRATE	0,85	008,45	CALCIUM CARBONATE	0,75	007,53
ROCK SALT	0,40	004,00	DICALCIUM PHOSPHATE DIHYDRATE	0,69	006,87
L-LYSINE HCL	0,07	000,75	ROCK SALT	0,40	004,00
CERDOS IB CREC-CEBO/F 0.2% NUT	0,20	002,00	SEPIOLITE	2,00	020,00
			HIGH-OLEIC SUNFLOWER OIL "A"	6,00	060,00
			L-LYSINE HCL	0,06	000,56
			CERDOS IB CREC-CEBO/F 0.2% NUT	0,20	002,00
